# STOPPING OLDER PERSON GENDER-BASED VIOLENCE IN WOMEN 55+ THROUGH PROMISING PRACTICES: A SCOPING REVIEW

**DOI:** 10.1093/geroni/igad104.3603

**Published:** 2023-12-21

**Authors:** Macri Melissa, Jihan Abbas, Sandra Hirst, Melanie Santarossa, Raza Mirza, Jessica Hsieh, Margaret MacPherson, Benedicte Schoepflin

**Affiliations:** Regional Geriatric Program of Toronto, Toronto, Ontario, Canada; Toronto Metropolitan University, Toronto, Ontario, Canada; University of Calgary, Calgary, Alberta, Canada; Baycrest Health Sciences, Toronto, Ontario, Canada; University of Toronto, Toronto, Ontario, Canada; University of Toronto, Toronto, Ontario, Canada; University of Western Ontario, London, Ontario, Canada; Canadian Network for the Prevention of Elder Abuse, Vancouver, British Columbia, Canada

## Abstract

**Background:**

Intimate partner and family violence (IPFV) disproportionately affect women of all ages, but are often understudied in older populations. Older women also face numerous barriers to reporting these types of abuse. A scoping review was undertaken to: (1) synthesize current knowledge about the utility and suitability of screening and intervention tools for older women (55+) experiencing IPFV; and (2) identify existing policy, practice, and research gaps with regard to current screening and intervention tools.

**Methods:**

A comprehensive search of the databases Medline, CINAHL, PsychINFO, AgeLine, ASSIA, and Sociological Abstracts was conducted. In addition, grey literature sources were searched using Google Scholar, ISI Social Sciences Citation Index, ISI Conference Proceedings Citation Index-Social Science & Humanities, Dissertations & Theses: Full Text, Canadian Institute for Health Information (CIHI), and National Institute of Health (NIH). After screening and selection, 42 documents were included for data extraction.

**Results:**

There were five major themes that emerged: (1) older women were not the specific targets of the studies/tools; (2) screening and intervention tools should address health outcomes; (3) tools identified were used or developed for some diverse populations; (4) two or more tools were used in combination; and (5) intervention tools should focus on social support and empowerment.

**Conclusions:**

Older women, especially those with intersectional identities, are rarely represented in studies of IPFV, and are even less frequently the primary focus. Screening and intervention tools should address health outcomes, social support, and empowerment. Effective screening and intervention may stem from utilizing multiple tools in combination.

